# Myocardial Infarction with Non-Obstructive Coronary Artery Disease: The Labyrinth of Investigations. Case Report and Review of the Literature

**DOI:** 10.3390/life11111181

**Published:** 2021-11-04

**Authors:** Paul Simion, Bogdan Artene, Ionut Achiței, Iulian Theodor Matei, Antoniu Octavian Petriș, Nicolae-Dan Tesloianu

**Affiliations:** 1 Interventional Cardiology Laboratory, Interventional Cardiology Compartment, Cardiology Clinic, Emergency Clinical Hospital “Sf. Spiridon”, Bd. Independenței nr. 1, 700111 Iași, Romania; paul-alexandru-smion@email.umfiasi.ro (P.S.); ionut-silviu.achitei@guest.umfiasi.ro (I.A.); iulian.matei@guest.umfiasi.ro (I.T.M.); dan.tesloianu@umfiasi.ro (N.-D.T.); 2 Intensive Care Unit Compartment, Cardiology Clinic, Emergency Clinical Hospital “Sf. Spiridon”, Bd. Independenței nr. 1, 700111 Iași, Romania; antoniu.petris@umfiasi.ro; 3 Department of Cardiology, “Grigore. T. Popa” University of Medicine and Pharmacy, 700115 Iași, Romania

**Keywords:** MINOCA, myocardial infarction, STEMI, myocardial resistance index, endothelial dysfunction, CMR

## Abstract

Myocardial infarction with non-obstructive coronary artery disease (MINOCA) accounts for approximately 5–15% of acute myocardial infarctions (MI). This infarction type raises a series of questions about the underlying mechanism of myocardial damage, the diagnostic pathway, optimal therapy, and the outcomes of these patients when compared to MI associated with obstructive coronary artery disease. We present the case of a 60-year-old patient with multiple cardiovascular risk factors and comorbidities who is admitted in an emergency setting. The patient is known with a conservatively treated inferior myocardial infarction which occurred 3 months prior, with reduced left ventricular ejection fraction. Emergency coronary angiography revealed normal epicardial coronary arteries, which led to further investigations of the underlying cause. Considering the absence of epicardial and microvascular spasm, CMR (cardiac magnetic resonance) confirmation of two transmural myocardial infarctions in the territories tributary to coronary arteries, and a high index of myocardial resistance in culprit arteries, we concluded the diagnosis of MINOCA due to the microvascular endothelial dysfunction. Although the concept of MINOCA was devised almost a decade ago, and these patients are an important part of MI presentations, it still represents a diagnostic challenge with multiple explorations required to establish the precise etiology.

## 1. Introduction

Myocardial infarction remains the leading cause of death and morbidity worldwide [1]. Although it is presumed that the absence of an artery blockage might imply a better prognostic, recent studies revealed that MINOCA patients tend to have the same or worse short-term prognosis compared to classic STEMI patients [2,3]. This can be explained by the difficulty in identifying a specific etiology and treatment. The European Society of Cardiology and the American Heart Association recommend advanced imaging techniques (intravascular ultrasound or optical coherence tomography, CMR, scintigraphy) for identification of a specific diagnosis for the tailoring of a targeted treatment [4,5].

We are reporting an atypical STEMI presentation and step-wise clinical and imagistic decisions aiming to elucidate an obscure endothelial dysfunction in the myocardium microvasculature, which eventually leads to transmural infarction tributary to epicardial territories.

## 2. Case Report

A 60-year-old male with multiple cardiovascular risk factors and comorbidities (chronic inferior myocardial infarction, stage 3 hypertension, type 2 diabetes, mixt dyslipidemia, sedentary life style, smoking) was admitted to our hospital in an emergency setting for recurrent angina pectoris that first occurred three days prior. His medical history revealed an inferior myocardial infarction 3 months prior, documented by the presence of q waves in the inferior leads on electrocardiography (ECG), inferior-wall hypokinesia, and global left ventricular ejection fraction (LVEF) of 35%. In the previous admission, the patient was treated medically due to his late hospital presentation and refusal to undergo coronary angiography. His chronic medical regimen consisted of: atorvastatin 10 mg o.d., aspirin 75 mg o.d., perindopril 10 mg o.d., bisoprolol 5 mg o.d., and metformin 1000 mg o.d.

Upon admission to our hospital, the patient was stable (BP = 160/82 mmHg, HR 105 bpm, oxygen saturation 99%), with mild bilateral basal crackles, and had severe recurrent angina pectoris (described as similar to the previous anginal episode).

**Figure 1:**
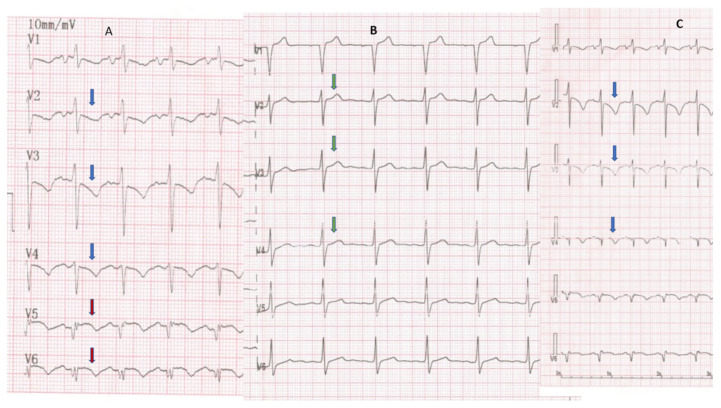
Intermittent ST-T ischemic changes (**A**): Anterior ST segment depression (blue arrows) with ST segment elevation in lateral leads (V5-V6) (red arrows) at admission. (**B**): Normalization of ST-T (green arrows) after 10 min. (**C**): ST segment depression in the anterior leads (blue arrows) during echocardiography.

**Figure 2:**
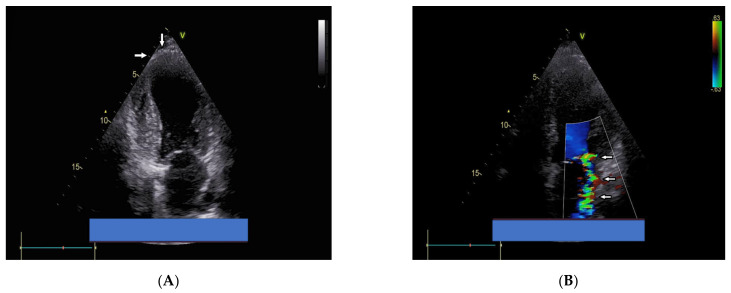
Admission echocardiography. (**A**): Apical left ventricle aneurysm (white arrows). (**B**): Secondary mitral regurgitation with eccentric jet due to the posterior cusp restriction (white arrows).

ECG revealed sinus rhythm, intermittent ST segment elevation in the anterolateral leads (V4–V6), ST segment depression in anteroseptal leads (V1–V3), and q waves in inferior (DII, DII, aVF) and lateral leads (DI, aVL, V5–V6) (Figure 1). High-sensitive troponin level was 1500 ng/L (normal range 0–29 ng/L), with dilated left ventricle (end systolic diameter of 58 mm), inferior, antero-apical, and anterolateral wall hypokinesia, apical aneurysm (Figure 2A; see Supplementary Video S1) resulting in an LVEF of 25% (calculated using Simpson), suggestive of severe systolic dysfunction. In addition, the right ventricle was mildly dilated with severe systolic dysfunction (fractional area change 20%, tricuspid annular plane systolic excursion 14 mm, S’ 7 mm/s on tissue Doppler). The mitral valve was thin, with normal opening, but with restriction of the posterior valve at closure resulting in a moderate secondary mitral regurgitation with eccentric jet following the lateral wall of the left atrium (proximal isovelocity surface area radius 8 mm, vena contracta 7 mm, effective regurgitant orifice area 0.2 cm^2^ with no pulmonary vein systolic flow reversal) (Figure 2B; see Supplementary Video S2). The aortic valve was tricuspid, thin, with normal opening and closure. There were no indirect signs of pulmonary hypertension, intracavitary formations, or congenital malformations. Considering the investigation, the diagnosis of STEMI was made and cath lab was activated. The patient received a loading dose of ticagrelor (180 mg), aspirin (300 mg), 80 mg of statin, and an iv infusion of NTG.

**Figure 3:**
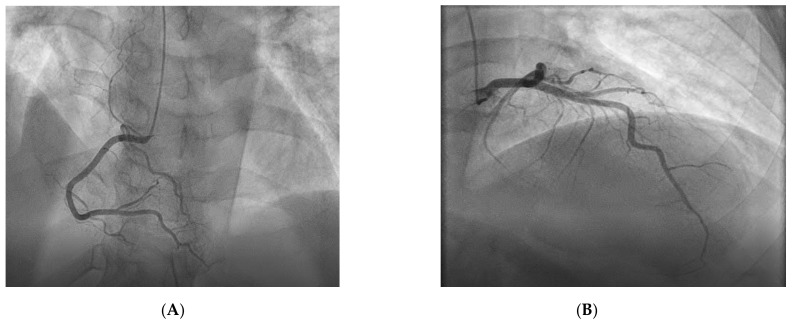
Emergency coronarography. (**A**): Normal right coronary artery. (**B**): Normal left coronary artery.

Coronary angiography revealed normal coronary arteries, with no angiographic signs of embolus, dissection, or unstable plaques (Figure 3A; see Supplementary Video S3; Figure 3B; see Supplementary Videos S4–S6). We concluded the diagnosis of MINOCA, and we continued with a step-wise approach in order to elucidate the etiology.

Considering the transient ECG ischemic changes (ST segment deviation, T wave inversions in the anterolateral leads), and new systolic wall motion abnormalities, severe vasospastic angina was suspected. A calcium channel blocker (Amlodipine 10 mg o.d.) and long-acting nitrates were added to the treatment schedule. After 5 days and a significant decrease in hs-Troponin level (50 ng/dL) and a 48 h prior cessation of vasodilator agents, we performed an intracoronary vasoreactivity test using ergonovine. The protocol was performed according to the JCS Joint Working Group Guidelines for Diagnosis and Treatment of Patients With Vasospastic Angina (JCS 2013) [6]. Baseline coronary angiography was performed without nitroglycerin usage. Slow infusion (course of 1 min) of 90 mcg of ergonovine for the left coronary artery and 60 mcg of ergonovine for the right coronary artery was performed, without chest pain, ECG changes, or epicardial spasm at 1, 3, 5, 7 min control contrast injections. The test was completed with administration of 200 mcg nitroglycerin in each coronary artery. Given the fact that was no criteria was fulfilled, the test was interpreted as negative. Positive criteria for vasoreactivity test require: (1) epicardial stenosis of > 90% following ergonovine injection; (2) angina after ergonovine injection with remission after nitroglycerin or spontaneously after 10 min; (3) ischemic ST-T changes following ergonovine injection [6]. According to the expert consensus on ischemia with non-obstructive coronary arteries, published in 2020 in collaboration with the European Society of Cardiology [7], if angina and ECG changes with no evident epicardial spasm are present, microvascular spasm is confirmed. In our case, the test was negative for epicardial and microvascular spasm.

**Figure 4:**
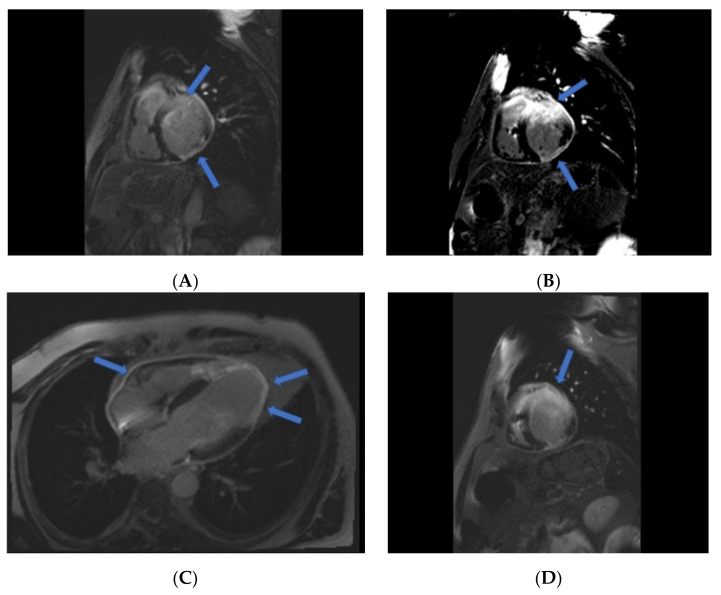
CMR. (**A**): Transmural subacute anterior wall infarction (blue arrows) and fibrotic transmural infero-lateral wall infarction. (**B**): Interstitial edema in the anterior basal septum and basal anterior segment (blue arrow). (**C**): Transmural infarction of the apical septum, apex, and anterolateral apical segment and epistenocardic pericarditis suggestive of Dressler Syndrome (blue arrow). (**D**): Extension of the interstitial edema in the anterolateral basal segment (blue arrow).

Considering the current findings with the exclusion of the vasospastic component, differentiation between true myocardial infarction and atypic myocarditis is mandatory. We recommended CMR, which revealed fibrotic transmural inferior wall infarction, subacute anterolateral, and apical wall infarction with interstitial edema, apical aneurysm, 25% LVEF, and epistenocardiac pericarditis suggestive of Dressler syndrome (Figure 4; see Supplementary Videos S7 and S8).

The result of the CMR excluded myocarditis and confirmed the transmural infarctions. Considering the normal angiographic coronary arteries and negative vasoreactivity test, we concluded that the endothelial dysfunction leading to a myocardial capacitance dysfunction is the true etiology of this atypical STEMI. This dysfunction can be explained by the capillary rarefaction and arteriolar atherosclerosis with microthrombi forming into a microvascular territory tributary to an epicardial coronary artery, mimicking an arterial blockage (STEMI).

For confirmation, we calculated the indirect hyperemic myocardial resistance index (HMR) using FFR in all three arteries and indirect measurement of coronary flow reserve (CFR) using transthoracic echocardiography doppler of proximal LAD. For obtaining FFR measurements we used the Phillips FFR probes and we performed the calculations after 200 mcgs of bolus intracoronary adenosine injection. The FFR was normal in all epicardial arteries. We calculated the hyperemic myocardial resistance using the formula:

*R* = *Pd*/*Q*

where *R* is HMR, *Pd* = distal pressure of coronary artery at maximal hyperemia, and *Q* is the flow derived from the transthoracic Doppler of the left main or LAD.

We obtained a value of 4, suggestive of increased microvascular resistance according to the European consensus in the treatment of coronary microvascular dysfunction published in 2020 [8]. Although myocardial microvascular dysfunction was established as an exclusion diagnosis, the confirmation using novel techniques of high microvascular resistance with no spasm component made a tailored medical treatment more feasible.

We introduced specific heart failure treatment: sacubitril/valsartan 49/51 mg o.d., carvedilol 6.25 mg b.i.d., ivabradine 2.5 mg b.i.d., dapagliflozin 5 mg o.d., and furosemide 40 mg o.d. for symptom control. We added amlodipine up to 10 mg o.d. for blood pressure control, a high-dose statin (80 mg o.d. atorvastatin), and 75 mg o.d. aspirin.

According to ESC Guidelines for the Management of Patients with Ventricular Arrhythmias and the Prevention of Sudden Cardiac Death, we decided to implant a single chamber ICD (Medtronic Mirro CMR^TM^ VR SureScan^TM^ DVME3D4, Medtronik, Inc., Minneapolis, MN, USA) for primary prevention of sudden cardiac death [9]. An RV electrode (Medtronic Sprint Quattro Secure S 6935 M) was inserted via the left subclavian vein and positioned at the apex of the right ventricle (RV). The postoperative examination on the first day was uneventful (no pericardial effusion and no pneumothorax). Stimulation thresholds for the RV electrode after implantation were 3.5 V/0.4 ms, with sensing at 3.5 mV. The patient was discharged after 7 days, being hemodynamically stable (BP 120/90 mmHg, HR 68 bpm), with NYHA II dyspnea, normal renal function (creatinine clearance of 90 mL/min/1.73 m^2^), and 25% LVEF with the recommendation to commence the cardiac rehabilitation program.

After 6 months, the routine evaluation revealed an improved exercise tolerance (the patient was walking in the range 13,000–15,000 steps/day, monitored using smartphone telemetry), with NYHA I dyspnea, 30% LVEF with persistent regional wall abnormalities, controlled BP (130/90 mmHg), HR 64/min, and no signs of ischemia, ventricular arrhythmias, and atrial fibrillation on the 24 h Holter ECG monitoring. The lipidemic profile revealed mild elevation of LDL-cholesterol levels (75 mg) and normal triglycerides values (80 mg). We added 10 mg/day of ezetimibe in order to reach the target LDL cholesterol.

This research was performed in accordance with the Declaration of Helsinki of 1975, revised in 2013. After extensive consultation, the patient gave verbal and written informed consent and fully authorized the authors to use his medical data for research purposes, as stated in the “Patient Informed Consent” (Order 1410/2016, issued by the Romanian Ministry of Health), signed by the patient.

## 3. Discussion

MINOCA represents an umbrella term for all the etiologies which can result in myocardial infarction in the absence of an arterial blockage. According to the European Society of Cardiology and American Heart Association, the definition requires: (1) acute myocardial infarction criteria: chest angina, ECG ischemic changes, cardiac imaging (myocardial perfusion imaging, CMR, or echocardiography) confirming new regional wall abnormalities, increased cardiac cytolysis biomarkers; (2) the absence of >50% epicardial stenosis at coronary angiography; and (3) no clinically overt cause for acute myocardial infarction presentation [4,5]. Given the many possible etiologies, MINOCA represents a diagnostic conundrum and usually requires multiple investigations.

Current studies suggest that between 5% and 15% of acute coronary syndrome presentations are diagnosed as MINOCA [1,3,10]. The term was implemented in the latest Fourth Universal Definition of Acute Myocardial Infarction to describe all entities with myocardial damage in the absence of significant epicardial stenoses [11]. According to the 2020 European Society of Cardiology position paper, there is a distinction between “true” and false, “pseudo” MINOCA. The former includes coronary disorders (dissection, plaque disruption, coronary spasm, microvascular dysfunction, and coronary thrombus/embolus), whereas the latter consists of myocardial disorders (myocarditis, Tako-Tsubo, Kounis syndrome, cardiomyopathies) and extracardiac disorders (stroke, pulmonary embolism, sepsis, acute respiratory distress syndrome, end-stage renal failure) [7]. Given the many etiologies, the Dutch ACS working group proposed that MINOCA should not be considered as a true diagnosis, but rather a clinical dynamic working diagnosis that needs to be elucidated [10].

The largest MINOCA study up to date, enrolling almost 270,000 unique patients, revealed a well-defined pattern of MINOCA patients. According to this registry, compared to classic acute myocardial infarction, women were two times more prevalent (*p* < 0.001), and patients were older (*p* < 0.001) (mean age 75 years) and with less conventional cardiovascular risk factors (8.7% vs. 1.3%, *p* < 0.001) [1].

Prognostic data regarding MINOCA are conflicting and vary with the underlying etiology. Currently, four major studies investigated this particular condition: SWEDEHEART [12], ACUITY [11], ACTION [13], and VIRGO [14], with contradictory results. MINOCA was associated with a lower mortality rate at 1 year in the ACTION (1.1% vs. 2.1%) and SWEDEHEART trials, whereas in the VIRGO study MINOCA was associated with the same rate of mortality at 12 months as the classic ACS patients (0.6% vs. 2.3%, *p* = 0.68) [11,12,13,14]. In the latter trial, patients were younger (<55 years) and had a similar profile as the Dutch ACS working group trial: less conventional cardiovascular risk factors (*p* < 0.001) and dominant female prevalence (*p* < 0.001) [14]. In the ACUITY trial, a total of 448 patients presenting with non-ST elevation myocardial infarction were included in the study (117 MINOCA and 331 with obstructive coronary disease), with no significant different baseline characteristics between the groups. Contrary to other studies, ACUITY revealed a higher 1-year mortality rate in the non-obstructive coronary artery disease group (5.2% vs. 1.6%, *p* = 0.04, 95%CI 1.05–11.28). Daniel et al. suggested that the results are explained by the lack of targeted treatment in MINOCA patients, compared to the atherosclerotic coronary artery disease where revascularization is the main therapeutic goal. Also, the study revealed that patients with nonobstructive coronary disease have a higher adjusted risk of mortality mainly from non-cardiac causes [12]. Although in the SWEDEHEART trial the mortality risk was higher in classic ACS patients, in the MINOCA subgroup the main cause of mortality was non-cardiac (58%).

Nathaniel R et al. in the ACUITY trial observed that 25% of MINOCA patients experienced angina in the following 12 months, and the presence of ST segment elevation, cardiogenic shock, and heart failure are more associated with mortality than the classic STEMI [13]. In our case, the patient had two transmural infarctions confirmed by CMR at 3 month intervals, and after optimal medical treatment at 6 months, he had no angina.

Jacqueline E. et al. propose in a 2019 MINOCA paper a step-wise diagnostic approach: (a) the confirmation of myocardial infarction and the absence of obstructive coronary disease based on clinical examination, transthoracic echocardiography, angiography, and elevated troponin levels. In this step, exclusion of alternative diagnostics such as sepsis, pulmonary embolism, cardiac contusion, etc., should be done. (b) “Working diagnostic step” consists of reviewing the angiographic films (to exclude missed lesions, embolus, coronary dissections) and excluding the myocardial injury due to nonischemic causes: Tako-tsubo (transthoracic echocardiography, ventriculography, CMR) and myocarditis (CMR). (c) Confirmation of MINOCA through intravascular imaging (exclusion of unstable plaques, emboli, intraluminal thrombi, dissection), coronary vasoreactivity test (epicardial, microvascular, or mixt spasm), and coronary functional assessment (FFR, iwFR, myocardial resistance index, hyperemic myocardial resistance index) [15].

Of importance is the differentiation between the microvascular dysfunction endotypes. In 2018 TJ Ford et al., in the CorMicA trial, described four types of microvascular dysfunctions: increased microvascular resistance (myocardial resistance index >25), decreased coronary vasorelaxation (coronary flow reserve <2.0), decreased microcirculation vasodilator capacity (resistive reserve ratio <2.0), and microvascular spasm (intracoronary vasoreactivity tests). This differentiation permits a tailored medical therapy (e.g., in microvascular spasm there is a calcium antagonist and long-acting nitrate approach, whereas, in increased microvascular resistance beta-blockers and ACE inhibitors are key therapy) [16]. This targeted therapy was implemented in the following European Society of Cardiology Consensus of microvascular dysfunction published in 2020 [7]. In our case we discovered an increased hyperemic myocardial resistance in the microvasculature, confirming an obscure endothelial dysfunction.

**Figure 5:**
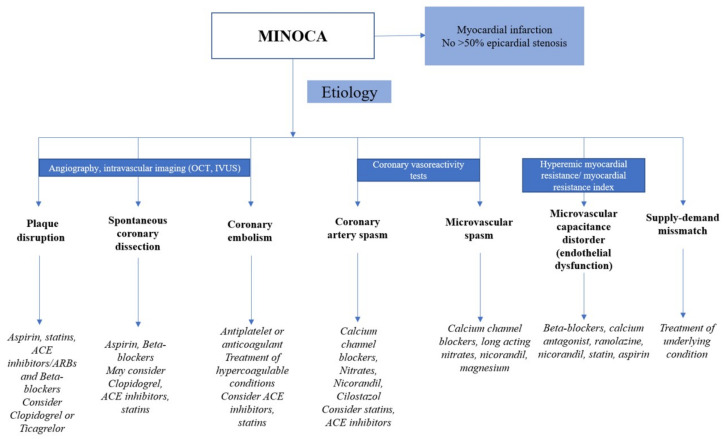
Treatment options for MINOCA. Adapted from Mukherjee D. et al., 2019 and Cormica study [16,17].

A schematic approach of the targeted treatment can be viewed in Figure 5.

## 4. Conclusions

Although the concept of MINOCA was devised almost a decade ago, and that MINOCA patients represent an important part of acute coronary syndrome presentations, there is still scarce evidence-based data for guiding the evaluation and management of these patients. Most of the MINOCA patients have fewer traditional cardiovascular risk factors, are younger and more frequently female, but still have similar or worse short-term prognosis compared to classic STEMI patients. To address its diagnostic and treatment complexity, an algorithm-based approach is suggested in the latest consensus paper’s recommendations. This review addresses the latest guidance on the subject, from the diagnostic work-up to the medical therapy of each constituent clinical entity. The key element of our case report is the step-wise diagnostic approach used to elucidate the underlying cause of a MINOCA patient (first confirming the transmural infarction, and after that finding the etiology using novel methods: FFR, derived microvascular resistance index, and intracoronary vasoreactivity tests).

## Data Availability

The data presented in this study are available on request from the corresponding author. The data are not publicly available due to privacy.

## Supplementary Materials

The following are available online at https://www.mdpi.com/article/10.3390/life11111181/s1, Video S1. Transthoracic two-dimensional echocardiography apical four-chamber view: Apical left ventricle aneurysm; Video S2. Transthoracic two-dimensional echocardiography apical four-chamber view: Secondary mitral regurgitation with eccentric jet due to the posterior cusp restriction. Video S3. Coronary angiography—right coronary artery (LAO cranial); Video S4. Coronary angiography—left coronary artery (LAO cranial); Video S5. Coronary angiography—left coronary artery (RAO caudal); Video S6. Coronary angiography—left coronary artery (LAO caudal);Video S7. Cardiac magnetic resonance: Transmural infarction of the apical septum, apex, and anterolateral apical segment; Video S8. Cardiac magnetic resonance: Transmural subacute anterior wall infarction and fibrotic transmural inferolateral wall infarction.
